# Nigeria and Italy Divergences in Coronavirus Experience: Impact of Population Density

**DOI:** 10.1155/2020/8923036

**Published:** 2020-05-21

**Authors:** Emmanuel O. Amoo, Olujide Adekeye, Adebanke Olawole-Isaac, Fagbeminiyi Fasina, Paul O. Adekola, Gbemisola W. Samuel, Moses A. Akanbi, Muyiwa Oladosun, Dominic E. Azuh

**Affiliations:** ^1^Demography and Social Statistics, College of Business and Social Sciences, Covenant University, Ota, Ogun State, Nigeria; ^2^Department of Psychology, College of Leadership Development Studies, Covenant University, Ota, Ogun State, Nigeria

## Abstract

**Background:**

The reports and information on coronavirus are not conspicuously emphasising the possible impact of population density on the explanation of difference in rapid spread and fatality due to the disease and not much has been done on bicountry comparisons.

**Objective:**

The study examined the impact of population density on the spread of COVID-19 pandemic in two sociodemographic divergent countries.

**Methods:**

The study conducted a scoping review of published and unpublished articles including blogs on incidences and fatalities of COVID-19. The analysis followed qualitative description and quantitative presentation of the findings using only frequency distribution, percentages, and graphs.

**Results:**

The two countries shared similar experience of “importation” of COVID-19, but while different states ordered partial lockdown in Nigeria, it was an immediate total lockdown in Italy. The physician/patient ratio is high in Italy (1 : 328) but low in Nigeria (1 : 2500), while population density is 221 in Nigeria and 206 in Italy. Daily change in incidence rate reduced to below 20% after 51 and 30 days of COVID-19 first incidence in Italy and Nigeria, respectively. Fatality rate has plummeted to below 10% after the 66^th^ day in Italy but has not been stabilised in Nigeria.

**Conclusion:**

The authors upheld both governments' recommending measures that tilted towards personal hand-hygienic practices and social distancing. Authors suggested that if Italy with its high physician/patient ratio and lower population density compared to Nigeria could suffer high fatality from COVID-19 pandemic under four weeks, then Nigeria with its low physician/patient ratio and higher population density should prepare to face harder time if the pandemic persists.

## 1. Introduction

As Nigeria was grappling with the challenge of high level of insurgency besetting certain parts of her territory in early 2000s, the devastating blow from Ebola pandemic (2014) came in and almost grounded her economy but with the proactiveness of her healthcare system that quickly doused the tension which culminated in declaration of the country free of Ebola by October 2014 [1, 2]. Ebola virus ravaged more than 11000 lives in Nigeria's neighbouring countries mainly in Liberia, Sierra Leone, and Guinea [3, 4]. It adversely affected communities and cities such as Conakry and Macenta (in Guinea); Kailahun, Kenema, Bombali, Port Loko, Western Rural and Western Urban (in Sierra Leone); Montserrado in Liberia, to mention but few [5, 6], according to the Centers for Disease Control and Prevention [7, 8]. However, as the country is scouting for strategies to attain the goals of sustainable development and Agenda 2063, in the face of fighting against incessant and highly reported serial killings, the communal conflicts, and herdsmen-farmers clashes [9–12], Lassa fever crept in [13]. Currently, the country is engulfed in a big fight for protection of lives under the mystifying coronavirus (COVID-19) that just reared up its ugly head [14–16].

Italy also, has in the last few years been faced with turbulence of severe landslides, floods, and bridge collapse [17–19] and is currently facing the public health challenge of COVID-19 pandemic. Cumulative casualties due to landslides, floods, and other recurring environmental challenges in Italy from retrospective data analysis was reported to be 50,593 comprised of dead, missing, or injured persons [20, 21]. As at March 31, 2020, Italy has recorded the highest death rate due to COVID-19 among other countries of the world. The public concern now is due to the alarming levels of spread and the fatality across the globe and the general question is how can this be quickly curtailed? The study analyzed whether sociodemographic divergency (such as population density) could have an impact on the spread of COVID-19.

Generally, the propensity for disease transmission is higher among the people that live in close proximity [22, 23]. Human population density is the number of people per unit of area, usually quoted per square kilometer (or square mile) which may include or exclude water areas or glaciers, denoted as population density=total population/land area in square km . Although the nature of the population density (which could be low or high) may not be a direct determinant of rapid spread of infections, there is high propensity for a densely populated area to become overcrowded, which could spur challenges in sanitation and declined quality of living conditions and potentially serves as breeding venue for infectious agents and rapid transmission [22–25]. When emergency cases therefore arise, the ease of curing the disease and the health system in addition to distribution of healthcare materials are often strained.

The name coronavirus is derivative of the Latin word “corona” that means “crown.” Biologically, they are named coronaviruses because they have spiky projections on their surface that look like crowns [26, 27]. The virus that is responsible for coronavirus disease belongs to the genus *Betacoronavirus* that cause several respiratory illness and other symptoms such as pneumonia, fever, breathing difficulty, and lung infection. Severe acute respiratory syndrome coronavirus 2 (SARS-CoV-2) is the virus strain that causes coronavirus disease (called COVID-19) [28, 29]. It is colloquially called coronavirus, previously referred to as 2019 novel coronavirus (2019-nCoV). There are diverse types of coronaviruses, such as SARS-CoV (the severe acute respiratory syndrome that was first identified in 2003), MERS-CoV (the virus that caused Middle East respiratory syndrome, first discovered in 2012), and the new SARS-CoV-2 that causes COVID-19 [26, 27, 30]. Specifically, COVID-19 was first identified in Wuhan City, Hubei Province, in China on December 29, 2019 [31–33]. World Health Organisation officially reported coronavirus on December 31, 2019, and by March 11 the disease was declared as a pandemic [34, 35], and it has since remained a public health emergency of international concern. Coronavirus incubation period is indicated to be 14 days, and median time from onset of the symptoms to intensive care unit admission is relatively 10 days, but the time between the onset and death is 2–8 weeks [27, 36–39].

Several factors have been suspected as the root cause of the spread of COVID-19, which include but not limited to lack of awareness, close contact with infected people, and touching eyes, nose, and mouth with contaminated hands [36–40]. Others include low hygiene behavioural practices and bilateral relationships between and among countries that permit cross-border travelling. Considering all these reports and information on coronavirus till date, there is no much emphasis on population density and major bicountry comparison on why the fatality is rapid and high in one country but low in another. This review is to provide additional information on the spread of the COVID-19 pandemic and increase the awareness on measures to curb the spread, and it also serves as an additional relevant resource to already existing literature on the infectious virus and its rapid spread.

## 2. Coronavirus in Italy and Nigeria: The Connections

The ties between Nigeria and Italy are rooted in decades of bilateral businesses where Italians establish business (companies) in Nigeria and Nigerians live and work in Italy, with each party paying dynamic attention to and respecting the rules, regulations, and sovereignty of each country. Nigeria has her embassy in Rome while Italy has the same and a consulate in Lagos. The two countries trade and exchange materials from leather to plastics and packaging, pharmaceuticals, building materials, training, intelligence sharing, and logistics supply, including technology and establishment of Italian Trade Agency (ITA) in Lagos State [41]. The relationship between the two countries has been peaceful and constructive, and there has not been any conspicuous trait that the tie may nose-dive or possibly collapse.

However, coronavirus in Nigeria could be linked to the international relationship between Italy and Nigeria that permitted cross-border travels between the two countries. The two countries had a similar first experience of coronavirus as an “imported disease” because it did not originate from either of the countries. As the pandemic sneaked into Nigeria through an Italian national, it also entered Italy via two tourists from China. Specifically, the two Chinese tourists (in Italy) were tested positive in Rome on January 31, 2020 [42, 43]. The third confirmed case in Italy was a repatriated Italian from the city of Wuhan (China) barely a week after the first two index cases were identified [28, 43]. The disease then started invading the nooks and crannies of Italy starting from a cluster of 16 cases in Lombardy, which later increased to 60, to the record of the first COVID-19 death in February 22, 2020 [28, 43]. Since then, the case-fatality rate has been very high and currently (as at March 31, 2020) accounting for almost one-third of the global deaths.

Weeks after the disease has been taking its toll on Italy, the disease started in Nigeria. The first reported index case of coronavirus disease in Nigeria was an Italian citizen in Lagos who tested positive to SARS-CoV-2 on February 27, 2020. The second case was reported on March 9, being the Nigerian contact of the first index case at the destination the infected Italian visited. The first Nigerian case was also the first case of the coronavirus in sub-Saharan Africa. Thereafter, other countries in the region have since recorded confirmed cases of COVID-19.

Nigeria is the largest and most densely populated country in Africa and the 7^th^ largest population in the world, with approximately 200 million people on a land mass area of 920,000 km (360,000 sq mi). Approximately more than 60% of Nigerians are urban dwellers, and the urbanization rate is estimated at 4.3%. Over 60% are younger than 25 years and the aged population is only 3.3% (Central Intelligence Agency) [44]. Italy, on the other hand, is a country of over 60 million people, and has 20 regions (regioni) divided into 110 provinces [45, 46]. It is one of the countries in Europe with a higher proportion of the aged. In Asia and Europe, the two continents that are home to the world's oldest populations (≥65 years), Japan shares 28% of the world aged population; Monaco, 26%; Italy, 23%; China, 12%; United States, 16%. However, India (in Asia) and Nigeria (in Africa) share only 6% and 3%, respectively (Population Reference Bureau) [47].

## 3. Materials and Methods

The study adopted a scoping review of published and unpublished articles including blogs covering updates on coronavirus incidences, deaths, and other related pandemic-health reports. For the data, population figures, density, and land area were extracted from 2019 world population data sheet, while data for coronavirus including the fatality and incidence cases, physician/persons, and so on were obtained using World Health Organisation Reports, NationMaster, World Bank Group, and worldometer. Certain information on daily occurrence of coronavirus in Italy and Nigeria was obtained from Dipartimento della Protezione Civile and Nigeria Center for Disease Control (NCDC). While the NCDC is Nigeria's national public health institute mandated to lead the preparedness, detection, and response to infectious disease outbreaks and public health emergencies, the *Dipartimento della Protezione Civile* is the only national body in Italy that is saddled with the responsibility of predicting, preventing, and managing emergency events such as national level disasters or catastrophes, both natural and human-made. The World Population Data Sheet, published by the Population Research Bureau (PRB), provides information on the key population, health, and environment indicators for more than 200 countries, and it is published annually. In terms of physician per 1000 person, the statistics available for Europe and Central Asia were used for the continent, respectively, while data for sub-Saharan Africa was imitated for Africa as a whole [48, 49].

We followed qualitative and quantitative descriptive analysis of the findings, reiterated the obvious consequences, and highlighted our assumed implications towards immediate solution to the spread of the epidemic and its further consequences. For the choice of the countries of study, we selected two countries with demographic variants. Life expectancy in the two countries are not the same. While Italy is the 5^th^ country in the world with highest life expectancy, 83.4 years (male = 81.1; female = 85.4) [50], Nigeria is one of the countries with lowest life expectancy rate with an average of 55.2 years (male, 54.7; female, 55.7) [51, 52]. The larger proportion of Nigerian population is young people (≤15 years), and the average age of the Italian population is 45.2 years and aged represent 21.7% of the Italian population [44].

## 4. Results

There are several reports on the incidences and fatality consequences of coronavirus. While only few are published, there is a lot of information on the coronavirus pandemic, which cannot be discarded in an emergency period like this. In an emergency period, every information should count. We reviewed several of these categories, sieved the reports where necessary, made a number of comparisons among different information, and extracted them for analysis. Although the information reviewed revealed that the topmost (as at 31 March 2020) in terms of COVID-19 incidence in Africa were South Africa (1326) and Egypt (609), Asia, China, Iran, and Turkey have recorded above 10,000 cases (China, 82241; Iran, 41495; Turkey, 11535) [53]. By April 26, 2020, Italy cases have gone up to 197,675 (Table 1), while Nigeria reported 1,273 cases (Table 1). However, the death tolls are highest in Italy (26,664) compared to other countries of the world and especially when compared with the death toll of 40 persons reported for Nigeria (Table 1).

The computations of percentage change in the incidences and fatalities rates from the compiled COVID-19 data for Italy and Nigeria are presented also in Table 1. The data were extracted mainly from *Dipartimento della Protezione Civile* (for Italy) and Nigeria Centre for Disease Control (NCDC) (for Nigeria), among others. The data covered only the time between the record of the first index case in the two countries and April 26, 2020. While the prevalence of COVID-19 has spanned 96 days in Italy, it has existed in Nigeria for 60 days at the time of this report.

Specifically, the incidences and fatalities from COVID-19 as at April 26, 2020 show that the incidence was staggering (at least) in the first three weeks in Nigeria. In Italy, the record was alarmingly galloping, crossed a thousand within the first six weeks of the incidence and increased to 10149 cases at exactly the 7^th^ week (March 10, 2020) from the first incidence at January 31, 2019 (Table 1). While COVID-19 was 12 days old in Nigeria before the country recorded the first death on March 9, 2020, there was no record of death in Italy until the 31^st^ day of COVID-19 in the country. However, at the time Nigeria was recording the first causality, Italy has recorded relatively half a thousand deaths (specifically, 463 persons). The result also revealed that the highest fatality percentage change (66.7–100.0%) was recorded for Italy around the 4^th^ week from the first index case (Table 1). In addition, the analysis revealed that Nigeria experienced the highest level of fatality (133.0%) at the 4^th^ week of incidence compared to Italy's experience around the same 4^th^ week from the first index case.

The declining rate for the incidence in Nigeria was remarkable in the first two weeks of April (≤10% on the average). We observed similar trends from Italy data where the incidence percentage change plummeted and remained below 5.0% in April. Both countries relatively experienced lower reduction in the incidence rates in April. The computation from the Nigeria data really shows that the incidence percentage change has not exceeded 15–20% for long range of weeks (precisely, throughout April).

**Figure 1:**
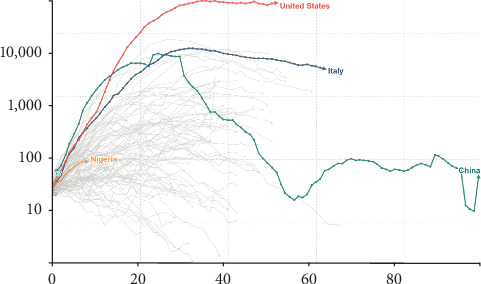
Daily confirmed COVID-19 cases (Nigeria, Italy, US, and China).

Granted that the dates of occurrence of COVID-19 in the two countries were not the same at the initial stage, records for concurrent data started on March 17, 2020. In other words, the daily confirmed cases continued chronologically from March 17 while the preceding dates witnessed a zigzag or spatial incidence level for both countries. Thus, we benched our graphical comparison from the date of the concurrent dataset for the two countries. Data for the graphical analysis therefore covered from March 17 to April 26, 2020.  Figure 1 represents the relative daily confirmed cases of COVID-19 for both Italy and Nigeria. This is simply the illustration of contribution of the two countries to the burden of COVID-19 in the global domain. Daily contribution from Nigeria is far below 100 cases while Italy is relatively above a thousand compared to the United States with a figure over 10000 as at April 26, 2020, while China that was previously high is currently below 100 (Figure 1). The graph depicts that the United States' incidence was far below China's and Italy's numbers at the onset but later outran other countries and now stands as the country with the highest incidence and fatality rates of coronavirus.

**Figure 2:**
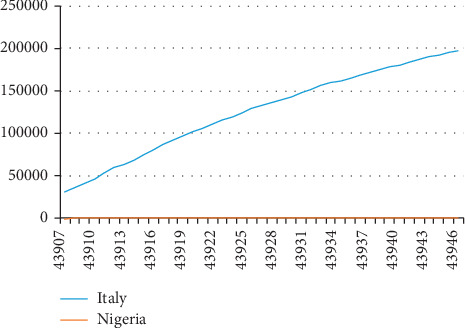
COVID-19 incidence in Italy and Nigeria (April 26, 2020).

**Figure 3:**
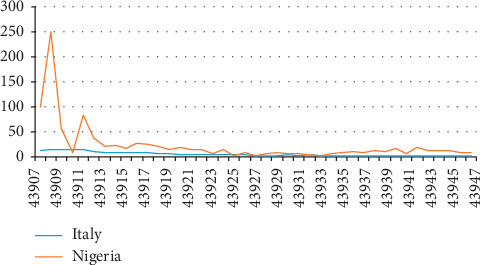
Percentage change in COVID-19 incident rates in Italy and Nigeria (April 26, 2020).

Figure 2 graphically shows the gap in the number of COVID-19 cases reported for Italy and Nigeria within the same period analyzed for the two countries and April 26, 2020. While, by comparison, the number reported for Nigeria is relatively low (hardly visible and parallel to the horizontal axis), it is positively sloping upward from left to right in respect of Italy.  Figure 3 illustrates the percentage change in the incidence rate of COVID-19 for the two countries in consideration and the relative distinctions in the movement of the curves for both countries. Nigeria's curve on the percentage change in incidence curve is observed to be negatively downward sloping from left to right, suggesting a reduction in the rate. However, the curve demonstrated a zigzag sloping as shown in Figure 3, and the percentage change for Italy was relatively consistently low.

**Figure 4:**
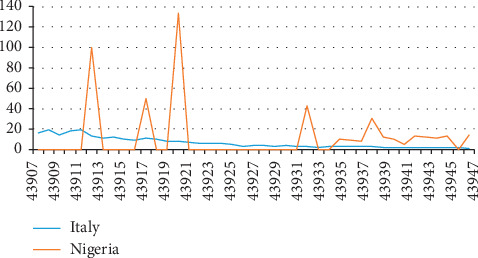
Percentage change in COVID-19 fatality rates in Italy and Nigeria (April 26, 2020).

The last figure (Figure 4) was used to demonstrate the percentage change in COVID-19 fatality rates between Italy and Nigeria. There is no doubt that the curves exhibited decline but a zigzag curve movement was also observed for Nigeria's curve, while it demonstrated a relatively steady decline for Italy.

However, the comparison between the selected demographic characteristics of the two countries shows relative divergences in population density per square kilometers, proportion of doctors per 1000 patients, and number of hospital beds per 10000 population. While the physician/patient ratio in Nigeria is 0.4 implying about one physician for 2500 patients, the ratio is 4.2 for Italy indicating one physician for 238 patients (Table 2). However, the ratio of number of hospital beds per 10000 population is 5.0 and 3.18 for Nigeria and Italy. respectively. The statistics is likened to 2,000 beds per 10000 population in Nigeria and 3,145 per 10000 population in Italy (Table 2). The population density that is calculated as the country area occupied divided by the total population shows variant results. While Nigeria population density per square km shows 221 human population per km^2^, it is 206 people per square kilometer for Italy (Table 2).

## 5. Current Measures and Precautions

Italy has placed all her population under self-quarantine. Other parts of the immediate measures taken by the Italian government have been immediate suspension of flights to and from China on 31 January 2019 with the closure originally meant for six months [54]. Italian government also introduced thermal scanners and temperature checks for all arriving passengers at the international Italian airports [54]. In addition, the government of Italy banned all movements except those on health emergencies and other special duties. In addition, more health facilities have been constructed between the time of the first case and now in both countries.

The specific trending measures are washing of hands, cleaning and disinfecting surfaces, and avoiding close contact with others. Considering the fact that part of the African culture is greeting and exchange of pleasantries, the World Health Organisation has recommended the people adopt greeting system that include nodding, waving of hands, a bow, and other gestures that are devoid of physical contact [28] (United Nations Children's Fund) [55, 56]. The public has also been advised to avoid touching eyes, nose, and mouth with their hands. Hands can pick up viruses especially when it is used in touching many surfaces. Thus, the use of contaminated hands to rub the face, nose, mouth, or eyes can make the person vulnerable to the infection [28, 39, 55], in addition to practising good respiratory hygiene by covering the mouth and nose with disposable tissue or a handkerchief and washing such handkerchief or coughing and sneezing at bent elbows [28] (United Nations Children's Fund) [39, 55].

## 6. Discussion

The study relatively chronicled the postmillennial era challenges faced by Nigeria from insurgency, to Ebola, kidnapping, serial killings, and herdsmen/farmers clashes and now the Corona pandemic in the face of the challenge of meeting the goals of sustainable development. It also highlighted the various environmental challenges encumbering Italy. From the materials reviewed, it can be seen that coronavirus has no boundary and everyone could be vulnerable. Economically, it stampeded most sectors and was a shock in transportation and logistic businesses. Open markets (as commonly found in sub-Saharan Africa) and stores were locked down, and streets were deserted. Schools and colleges (both public and private) were closed. While salaries and allowances from the public enterprises could be guaranteed, the same cannot be held for the private business sector whose employee's take-home pay is a function of the generated income [57, 58]. Nevertheless, COVID-19 has created unity among nations; the lockdown, sit-at-home order has created and reinstated bounds within family and communities. Efforts to curb the spread defiled unicountry approach and created a multilateral unification among all stakeholders and the populace. Notwithstanding, our report could spur or serve as basis for future investigation on the postpandemic health status of the citizenry in both Nigeria and Italy.

The many problems of Nigeria and Italy could be responsible for their lack of preparedness towards the outbreak of disease. The preoccupation with quenching insurgency, communal clashes, and environmental problems could befog the reasoning for guiding against the utmost health consequences of these challenges. Health is population and population is health. Deficiency in health facilities exposes the country more to health disasters and often results in insufficient capacity to care for population where the need arises. The revelation of the low physician/patient ratio of 1 : 2500 in Nigeria and 1 : 328 in Italy signals danger for Nigeria, should the epidemic extend beyond the current level. If Italy with its high physician/patient ratio and lower population density compared to Nigeria could suffer as much fatality as experienced with ongoing incidences of COVID-19, then other countries with abysmally low level as seen for Nigeria should prepare to face harder time in disaster period.

Notwithstanding the above, as at the time of writing this report, the fatality rate in Nigeria has not increased sharply despite the low level of health facilities compared to Italy or other affected developed nations. Government response or perhaps inadequate testing facility could have accounted for this. As at the 31^st^ day of COVID-19 infection in Nigeria, the percentage incidence increase was 20% while Italy figure remained relatively ≤2 persons. However, around this period, when the disease has been present for relatively 10 weeks (precisely 68 days) in Italy and 60 days in Nigeria, the percentage incidence increase was 5.3% for Italy and 7.7% for Nigeria. While the change in fatality was 7.5% increment in Italy, it was 14.3% in Nigeria. Notwithstanding, the people's experience, the government, and the local public health officials' handling of the immediate past-Ebola outbreaks and ongoing monitoring of Lassa fever could have also played out [2, 13, 59].

Although the levels of proactiveness (or reactiveness) by the different governments were not measured in this study, the measures that were rolled out suggest urgent attention was devoted to the epidemic by the governments of the two countries. However, the total lockdown might not be absolutely benign. The challenge in food rationalization or purchase, the ensuing overcrowding including its immediate and long-run health effects, especially in Nigeria, where average family is large and all members could present at home, and the restrictive mass quarantine could create more anxiety at home and escalate incidence rate. In addition, the current situation could expose the inefficiency of the health system and the laxity and level of depravedness in the health systems of the two countries. The massive construction of new hospitals, intensive care units, and isolation centers are pointers to past omissions on citizens health at the level of government and such error should not be repeated in the future.

The observation that the incidence percentage change has not exceeded 20% (Nigeria) and 5.0% (Italy) in April seems to portend better possibility that the health systems is coping adequately with the disease. Also, Nigerian cases that were low at the onset could be (perhaps) due to the quick intervention by the government and alertness of the health system. However, since there is no immediate curative vaccine or definite cure for the disease currently, maintenance of the existing measures could be sustained.

Italy is a developed economy with high standard of living, higher employment rate, and good financial structure including excellent telecommunications. High proportion of people with infectious disease drains the reputation of any country and, if persistent, it could relegate such country to lower economy class. This could be a bad omen for the future stabilization of development of Italy if she continues to suffer from natural and environmental disasters including COVID-19. Similar fate awaits Nigeria if she should continue to experience human-made and natural turbulence like the current pandemic situation. Finally, recession could be inevitable if the pandemic and the lockdown should continue in these two countries.

## 7. Conclusion and Recommendations

The study outlined some similarities and divergences between Nigeria and Italy. It identified that both countries had similar antecedence of natural and human-made disasters and are currently experiencing COVID-19 pandemic but in different dimensions especially in terms of magnitude. The coronavirus entered the two countries following a similar pattern. However, demographically, Italy and Nigeria are two countries apart: while Nigeria is rich in younger population (≤15 years), relatively, the average age in Italy is 45.2 years. The study also identified the total lockdown as a major initiative of both Italian and Nigerian governments, though Nigeria lockdown came weeks after Italy has started. Recommended measures are generally tilted towards personal hand hygienic practices. However, since lockdown is already taking place in both countries and other nations, the authors suggest that economic digitalisation such as online businesses, e-learning, and e-governance that guarantee minimal human physical contact should be encouraged in Nigeria, Italy, and the rest of the world for plausible prevention and management of COVID-19 pandemic. In addition, closer monitoring and control of the pandemic spread in still unavoidable in Nigeria considering the current nonsteady decline in the fatality level.

## Figures and Tables

**Table 1:** Incidences and fatalities from COVID-19 in Italy and Nigeria as at April 26, 2020.

	Italy					Nigeria				
	Date from 1^st^ incidence	Cumulative cases	Cumulative deaths	Incidence % change	Fatality % change	Date from 1^st^ incidence	Cumulative cases	Cumulative deaths	Incidence % change	Fatality % change
1/31/2020	1st	2	0	—	—					
2/21/2020	31st	20	1	—	—					
2/22/2020	32nd	79	2	295.0	100.0					
2/23/2020	33rd	150	3	89.9	50.0					
2/24/2020	34th	229	6	52.7	100.0					
2/25/2020	35th	322	10	40.6	66.7					
2/26/2020	36th	445	12	38.2	20.0					
2/27/2020	37th	650	17	46.1	41.7	1st	1	—	—	—
2/28/2020	38th	888	21	36.6	23.5	2nd	—	—	—	—
2/29/2020	39th	1,128	29	27.0	38.1	3rd	—	—	—	—
3/1/2020	40th	1,694	34	50.2	17.2	4th	—	—	—	—
3/2/2020	41st	2,036	52	20.2	52.9	5th	—	—	—	—
3/3/2020	42nd	2,502	79	22.9	51.9	6th	—	—	—	—
3/4/2020	43rd	3,089	107	23.5	35.4	7th	—	—	—	—
3/5/2020	44th	3,858	148	24.9	38.3	8th	—	—	—	—
3/6/2020	45th	4,636	197	20.2	33.1	9th	—	—	—	—
3/7/2020	46th	5,883	233	26.9	18.3	10th	—	—	—	—
3/8/2020	47th	7,375	366	25.4	57.1	11th	—	—	—	—
3/9/2020	48th	9,172	463	24.4	26.5	12th	2	1	100	0
3/10/2020	49th	10,149	631	10.7	36.3	13th	—	—	—	—
3/11/2020	50th	12,462	827	22.8	31.1	14th	—	—	—	—
3/12/2020	51st	15,113	1,016	21.3	22.9	15th	—	—	—	—
3/13/2020	52nd	17,660	1,266	16.9	24.6	16th	1	1	−50	0
3/14/2020	53rd	21,157	1,441	19.8	13.8	17th	—	—	—	—
3/15/2020	54th	24,747	1,809	17.0	25.5	18th	—	—	—	—
3/16/2020	55th	27,980	2,158	13.1	19.3	19th	—	—	—	—
3/17/2020	56th	31,506	2,503	12.6	16.0	20th	2	1	100.0	0.0
3/18/2020	57th	35,713	2,978	13.4	19.0	21st	7	1	250.0	0.0
3/19/2020	58th	41,035	3,405	14.9	14.3	22nd	11	1	57.1	0.0
3/20/2020	59th	47,021	4,032	14.6	18.4	23rd	12	1	9.1	0.0
3/21/2020	60th	53,578	4,825	13.9	19.7	24th	22	1	83.3	0.0
3/22/2020	61st	59,138	5,475	10.4	13.5	25th	30	2	36.4	100.0
3/23/2020	62nd	63,927	6,077	8.1	11.0	26th	36	2	20.0	0.0
3/24/2020	63rd	69,176	6,820	8.2	12.2	27th	44	2	22.2	0.0
3/25/2020	64th	74,386	7,503	7.5	10.0	28th	51	2	15.9	0.0
3/26/2020	65th	80,539	8,215	8.3	9.5	29th	65	2	27.5	0.0
3/27/2020	66th	86,498	9,134	7.4	11.2	30th	81	3	24.6	50.0
3/28/2020	67th	92,472	10,023	6.9	9.7	31st	97	3	19.8	0.0
3/29/2020	68th	97,389	10,779	5.3	7.5	32nd	111	3	14.4	0.0
3/30/2020	69th	101,739	11,591	4.5	7.5	33rd	131	7	18.0	133.3
3/31/2020	70th	105,792	12,428	4.0	7.2	34th	151	7	15.3	0.0
4/1/2020	71st	110,574	13,155	4.5	5.8	35th	174	7	15.2	0.0
4/2/2020	72nd	115,242	13,915	4.2	5.8	36th	184	7	5.7	0.0
4/3/2020	73rd	119,827	14,681	4.0	5.5	37th	209	7	13.6	0.0
4/4/2020	74th	124,632	15,362	4.0	4.6	38th	214	7	2.4	0.0
4/5/2020	75th	128,948	15,887	3.5	3.4	39th	232	7	8.4	0.0
4/6/2020	76th	132,547	16,523	2.8	4.0	40th	238	7	2.6	0.0
4/7/2020	77th	135,586	17,127	2.3	3.7	41st	254	7	6.7	0.0
4/8/2020	78th	139,422	17,669	2.8	3.2	42nd	274	7	7.9	0.0
4/9/2020	79th	143,626	18,279	3.0	3.5	43rd	288	7	5.1	0.0
4/10/2020	80th	147,577	18,849	2.8	3.1	44th	305	7	5.9	0.0
4/11/2020	81st	152,271	19,468	3.2	3.3	45th	318	10	4.3	42.9
4/12/2020	82nd	156,363	19,899	2.7	2.2	46th	323	10	1.6	0.0
4/13/2020	83rd	159,516	20,465	2.0	2.8	47th	343	10	6.2	0.0
4/14/2020	84th	162,488	21,067	1.9	2.9	48th	373	11	8.7	10.0
4/15/2020	85th	165,155	21,645	1.6	2.7	49th	407	12	9.1	9.1
4/16/2020	86th	168,941	22,170	2.3	2.4	50th	442	13	8.6	8.3
4/17/2020	87th	172,434	22,745	2.1	2.6	51st	493	17	11.5	30.8
4/18/2020	88th	175,925	23,227	2.0	2.1	52nd	541	19	9.7	11.8
4/19/2020	89th	178,972	23,660	1.7	1.9	53rd	627	21	15.9	10.5
4/20/2020	90th	181,228	24,114	1.3	1.9	54th	665	22	6.1	4.8
4/21/2020	91st	183,957	24,648	1.5	2.2	55th	782	25	17.6	13.6
4/22/2020	92nd	187,327	25,085	1.8	1.8	56th	873	28	11.6	12.0
4/23/2020	93rd	189,973	25,549	1.4	1.8	57th	981	31	12.4	10.7
4/24/2020	94th	192,994	25,969	1.6	1.6	58th	1098	35	11.9	12.9
4/25/2020	95th	195,351	26,384	1.2	1.6	59th	1182	35	7.7	0.0
4/26/2020	96th	197,675	26,644	1.2	1.0	60th	1273	40	7.7	14.3

Sources: The Protezione Civile (2020); Nigeria Centre for Disease Control (NCDC) (2020); and other unpublished works.

**Table 2:** Selected demographics, health indicators, and COVID-19 incidences for Nigeria and Italy.

	Italy	Nigeria
Demographics		
Population	60,550,075	200,963,599
Land area (km^2^)	294,140	910,768
Population density (per km^2^)	206	221
Density (per mile^2^)	533	572
Selected health indicators		
Number of state/province	110	36
Hospital beds/10000 population	3.18	5.0
No. of physicians/1000 persons	4.2	0.4
Population growth rate	0.23	2.6
COVID-19		
Incidences	197,675	1,273
Deaths	26,644	40
Recovery	64,928	239

Sources: computed for this study from reviewed reports.

## Data Availability

The principal data used for this study are extracted from different sources as indicated below, and the computation used to support the findings of this study are included within the article. (1) Protezione Civile (2020), Protezione Civile Bulletin at 18 : 00 CET, The Dipartimento della Protezione Civile, Italy (http://www.protezionecivile.gov.it/web/guest/media-comunicazione/comunicati-stampa) (https://en.wikipedia.org/wiki/2020_coronavirus_pandemic_in_Italy). (2) Nigeria Centre for Disease Control (NCDC), March 31, 2020 (https://www.ncdc.gov.ng/diseases/sitreps/?cat=14&name=An%20update%20of%20COVID-19%20outbreak%20in%20Nigeria) . (3) Population Reference Bureau (PRB), The 2019 World Population Data Sheet, Population Reference Bureau (PRB), Washington DC (https://www.prb.org/worldpopdata).
